# Towards Improved Management of Tropical Invertebrate Fisheries: Including Time Series and Gender

**DOI:** 10.1371/journal.pone.0091161

**Published:** 2014-03-10

**Authors:** Sara Fröcklin, Maricela de la Torre-Castro, Elin Håkansson, Anna Carlsson, Madeleine Magnusson, Narriman S. Jiddawi

**Affiliations:** 1 Department of Ecology, Environment and Plant Sciences, Stockholm University, Stockholm, Sweden; 2 Department of Physical Geography and Quaternary Geology, Stockholm University, Stockholm, Sweden; 3 Stockholm Resilience Centre, Stockholm University, Stockholm, Sweden; 4 Institute of Marine Science, University of Dar es Salaam, Zanzibar, Tanzania; Leibniz Center for Tropical Marine Ecology, Germany

## Abstract

Invertebrate harvesting is an important livelihood in tropical settings providing income and food for numerous populations throughout the world. However, the lack of research, policy and management directed towards this livelihood hinders the analysis of time trends to evaluate invertebrate resources status. Another missing aspect is the consideration of gender analysis, i.e., the different roles and interests of men and women engaged in this activity. Based on interviews, catch assessments and inventories this multi-disciplinary study from Chwaka Bay (Zanzibar, Tanzania) shows how unregulated harvesting of invertebrates may result in sharp declines in animal abundance over a relatively short period of time (2005 to 2010), threatening the sustainability of the fishery. Specifically, the results show that catches in general, and prime target species of gastropods and bivalves in particular, had been significantly reduced in number and size. Interviews revealed gender disparities; female harvesters experienced less access to good fishing/collecting grounds and species of high value, which subsequently resulted in lower individual income. This is tightly linked to women's reproductive roles, which not only leads to limited mobility but also lessen their chances to accumulate livelihood assets (natural, physical, financial, social and human capital) thus impacting livelihood strategies. To protect invertebrate resources from overexploitation, and assure a constant flow of income and food for future generations, this case study illustrates the need for formal monitoring to assess changes in invertebrate resources, and possible ecological consequences, over time. Managers and policy-makers must also address gender to evaluate the contribution of all resource users, their capacity to cope with changing conditions, as well as specific interests.

## Introduction

There are many studies dealing with overfishing of coral reef fish in the tropics [1]; however, other fishing activities providing income and food sources are less studied. This is the case of invertebrate fisheries, which are rapidly expanding globally as a consequence of declining finfish fisheries [2]. Hypothetically, there may be several reasons behind this lack of research, policy and management attention directed to both environmental and social dimensions of invertebrate fisheries: First, the idea that invertebrate fisheries are particularly resistant to overfishing [3], second, the undervaluation of invertebrates as an important food source [2], and third, the typical view of the fishing industry as a men's sphere with focus on finfish fisheries and not on women-dominated invertebrate harvesting in near-shore areas [4]. This oversight, except for the economic valuable groups of lobsters, oysters and sea cucumbers [5]–[7], has resulted in poor catch documentation and statistics. In addition, data on invertebrate resources stock status is often limited [2], [8]–[9], which hinders analyses of time trends in species richness and abundance. Another growing concern is the lack of gender focus in fisheries; although data on women's role and contribution to fisheries has increased over the last years [4], [10] there is still a long way to go (e.g. [4], [11]–[12]). Underestimating the role both men and women play in relation to resource utilization may lead to resource threats being miscalculated. Further, it may overlook the social, cultural and political structures that determine the livelihoods of men and women [10]. The lack of attention given to these social and environmental aspects of invertebrate fisheries may, theoretically, jeopardize ecologically viable invertebrate populations and could have severe impacts on people's livelihoods.

To redress the lack of understanding about the effects of daily harvesting activities on invertebrate resources, as well as the gender dimensions associated with fishery, here we present a case study from Chwaka Bay, Zanzibar, Tanzania. The study was conducted at two occasions; one in 2005 and one in 2010, and shows how constant harvesting pressure by both males and females may reduce species richness and abundance and thus lessens the flow of marine goods and services crucial for coastal people. The study further analyzes how gender, as key societal organizer [13]–[14], affects the way in which men and women engage in this important livelihood. A common definition of a livelihood is that provided by Ellis [15] wherein a livelihood ‘comprises the assets (natural, physical, human, financial and social capital), the activities and the access to these (mediated by institutions and social relations) that together determine the living gained by the individual or household’. To make a comprehensive analysis we combined gender aspects, analyzing differences and similarities between males and females engaged in invertebrate collection, along with catch assessments and inventories of invertebrate resources. The working hypotheses in the study were i) that invertebrate abundance has decreased over a five year period due to high and chronic collection pressure, which in turn ii) affects catch composition (number of species and weight), and iii) that there are gender-based differences in access to fishing/collecting grounds and high-value species which in turn result in different individual income levels for male respective female harvesters. The findings from Chwaka Bay provide a wider understanding about the role of invertebrate resources as income and food sources, both for the individual collector and households, but also raise some concerns of potential ecological consequences as a result of constant fishing pressure and unregulated utilization. Equally important, it illustrates the role of both men and women in the fishery. The study contributes with crucial information about two important aspects for tropical invertebrate fisheries policy and management, i.e. the insight that invertebrate populations can decline relatively fast and that gender must be considered in the analysis, and should lead to more informed decisions in fisheries policy and management.

## Methods

**Figure 1:**
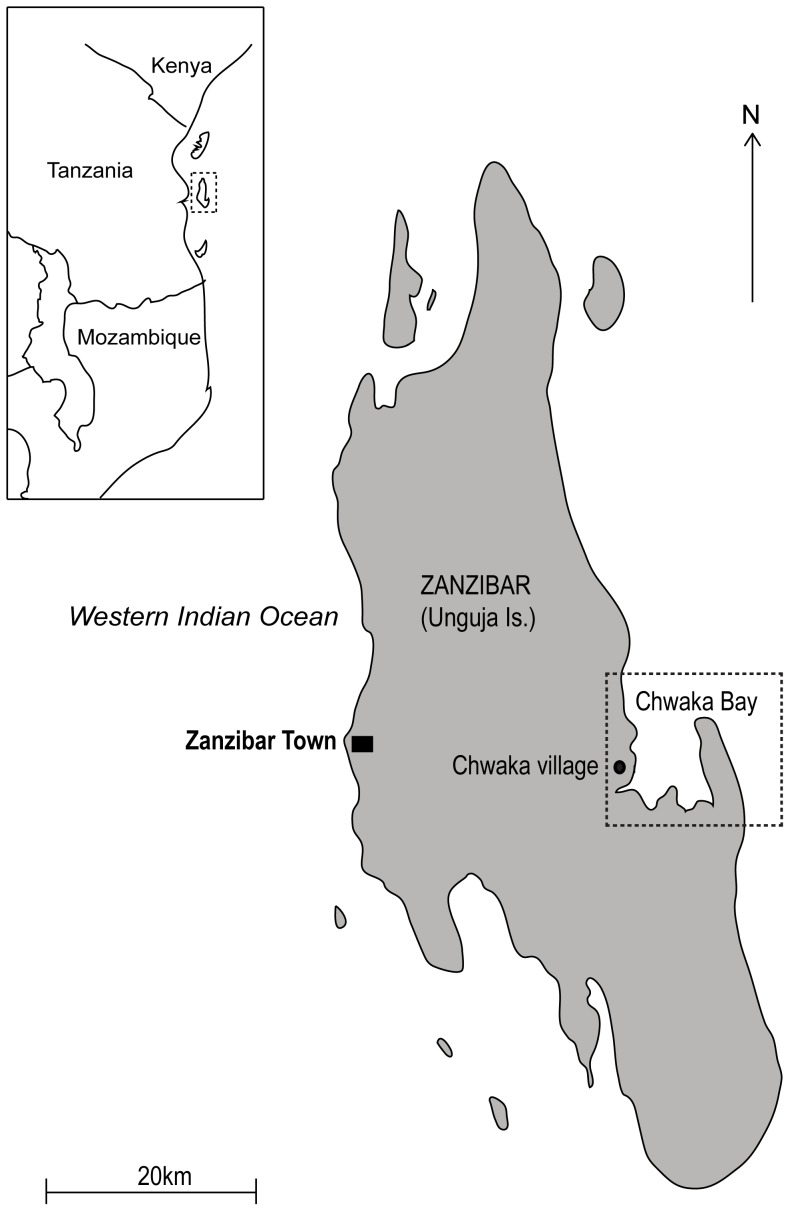
Study area Chwaka Bay, Unguja Island (Zanzibar), 6° 13′ 00″–02′54″ S and 39° 23′38″–32′00″ E. Interviews and catch assessment were conducted in Chwaka village, indicated by a filled circle. Biological field sampling was carried out on different sites in Chwaka Bay; the area being marked by a dotted line.

The research was undertaken in Chwaka Bay, located on the east coast of Zanzibar (Unguja Island) off the coast of mainland Tanzania (Fig. 1). Data was sampled at two occasions, one during March to June 2005 and the other from June to August 2010. In both sampling periods we conducted semi-structured and in-depth interviews with both male and female harvesters, catch assessments of invertebrates collected and biological inventories of invertebrates and substrate on the collecting grounds. In addition, the legal documents for Zanzibar fisheries management, such as the Fisheries Acts of 1993, 1998 and 2010, were analyzed.

### Ethics statement

Due to high illiteracy rates verbal consent from participants was obtained before conducting all interviews. The participants were informed about the purpose of the study and how the data would be utilized as well as secured anonymity, and only those giving consent were interviewed. A few under-aged respondents were interviewed (17 out of 150); the decision to interview adolescents was made based on their daily participation in collecting activities. Verbal consent was given by the parents and when no parents were present information about the study and anonymity was explained to the minor and only those giving consent were interviewed. The relatively long stay in the village enabled us to follow up respondents (particularly the minors) and make sure that potential questions or concerns related to the purpose of the study and use of information were clarified. The board of the Dept. of Ecology, Environment and Plant Sciences at Stockholm University approved the research plan and ethical aspects of the interview process. This board does not deal with ethical issues specifically; it rather approves research projects as a whole and assures that it follows ethical standards. In addition, the research was approved and research permits for all non-local researchers were obtained through the Institute of Marine Science (IMS) in Zanzibar as well as the Government of Zanzibar. Interviews with minors were not approved on beforehand but decisions concerning these interviews, as well as ethical considerations, were taken when facing the reality of the situation in the field.

### Study area

Chwaka Bay has an area of about 50 km^2^ and is surrounded by extensive mangrove forests and coastal vegetation [16]. It constitutes a mix of inter- and subtidal flats, seaweeds and is a hotspot for seagrass diversity with 11 species represented (among others, *Thalassia hemprichii*, *Cymodocea serrulata, Cymodocea rotundata, Enhalus acoroides* and *Thalassodendron ciliatum*) [17], which harbours great abundances of fish and invertebrates [16], [18]. Particularly seagrass beds are known to sustain high species richness [19]–[20]. The daily tidal range is 1 m for neap and 3.5 m for spring tide [21], and to navigate the Bay boat transport is often required [22]. Seven villages are located around the Bay and Chwaka village, where this research was conducted, is the largest with approximately 3 200 inhabitants [23]. The reason for working in Chwaka village was based on previous research and extensive knowledge in both social and ecological system dynamics [24], but also a result of time as well as economic constraints. Harvest of invertebrates such as gastropods, bivalves and octopus occurs on a daily basis and is, together with finfish fisheries and seaweed farming, one of the most important sources of food and income for local people [18], [25]. Up to 50 different species are collected [26]. Mollusc shells may further be used for limestone production, chicken food supplements [27], or as a trade product within the tourism industry [28]. The multiple use and markets available make invertebrates an important part of the livelihood portfolio disposable for Chwaka village's mainly low-income households.

### Interviews

To analyze differences in harvesting activities among male and female respondents a “gender analysis” was adopted [29]–[30]. For this analysis, interviews played a major role [31]–[32]. We carried out semi-structured interviews with males and females collecting invertebrates to solicit information about i) time spent on collecting, ii) fishing/collecting grounds and substrate used for collection, iii) targeted species, invertebrate use and individual daily income generated, and iv) specific needs and interests related to harvesting activities (see Text S1). Daily income data was obtained from interviews and based on the individual's own estimation. In 2005, 42 female (age 12–70) and eight male (17–55) harvesters participated in the interview study and in 2010, 40 female (age 15–65) and 40 male (age 15–68) harvesters answered the same interview form. The difference in the number of male respondents between years was due to time constraints and thus unwillingness to participate. To deepen the understanding about household structure, specific needs and challenges related to the activity, in-depth interviews with ten women (age 20–47) and ten men (age 18–37) were added to the data collection in 2010 (see text S1). We selected respondents on the basis of their active involvement in invertebrate harvesting, willingness and availability to participate in the study. The number of interviewees should be representative for the village; in 2005 all households with people involved in invertebrate harvesting (about 14% of all households in Chwaka village) were interviewed. In 2010, the number of people engaged in this activity had increased, thus, people from 80 unique households were interviewed. None of the female or male respondents were from the same households. The authors' great experience from working in this village allowed for the establishment of good communication. It also enabled us to follow harvesters during work, at the local market, and in their homes and opened up for informal discussions with middlemen (n = 4) based in Chwaka village about trade and market issues.

Both types of interviews (semi-structured and in-depth) followed a set of questions and are similar in the sense that they allow for flexibility; however, the in-depths interviews went further in the extent to which emphasis was placed on the respondents own thoughts about, not seldom, very complex issues about household structures [31]–[32]. Semi-structured interviews lasted for about 40 minutes whereas in-depth interviews generally lasted for more than an hour. All interviews were recorded for further analysis. Interviews were performed by the authors and with the assistance of local translators (several different translators were used due to the time span of the study) from the Institute of Marine Sciences (IMS). Price information on the particular invertebrate species was obtained directly from the monitoring agent (*Bwana Diko*) based in Chwaka village. No price information was collected for 2010, thus, to be able to make comparisons over time additional data on price was collected in 2013. The actual price was adjusted for inflation using annual average percentage change in consumer prices from the 2013 World Economic Outlook [33], following: Inflation adjusted price_n_ = Actual price_n_/Consumer Price Index_n_. The average inflation rate during this time span was 9.6%.

### Catch assessments

Daily catch data from ten females in 2005 (catches n = 107) and ten females in 2010 (catches n = 136) was analyzed for a 14-day period, i.e. one spring and one neap tide to capture a full tidal cycle. Again, due to time constraints of male fishers only 23 of their catches in 2005 and 22 catches in 2010 were analyzed, and no weight data was obtained in 2010. The authors analyzed all catches directly on the beach or at the marketplace in the village. Animals were identified to lowest taxonomic level possible and quantity and weight (wet weight including shell) was recorded [34]–[36].

### Biological inventories

We performed inventories of invertebrate populations during daytime at low-water spring tide (0–70 cm depth) to investigate the condition of invertebrate resources at 11 fishing grounds spread within the Bay where collection normally takes place (site descriptions are found in Table S1). The reason for conducting inventories during daytime at low tide is twofold; we wanted to conduct the sampling at the same time as people do their collection and the exposure to light also minimizes the risk of missing animals in the counts. The 11 sites were chosen on the basis of information derived from the collectors during interviews as well as personal observations by the authors regarding popularity and frequency of use. At each site 13–20 1×1 m steel frames were placed randomly and epibenthic invertebrates (>1 cm) were counted and identified to lowest taxonomic level possible [34]–[36]. In addition, benthic cover of seagrass, algae, sand and rocks (%) in each steel frame was visually estimated. A total number of 230 replicates in 2005 and 232 in 2010 were analyzed. Inventories were conducted at the same 11 sites both years to be able to make robust comparisons in animal abundance, species composition and benthic cover between the years.

### Statistical analysis

#### Interviews

We used time spent on collecting, fishing/collecting grounds, targeted species, and species use, daily individual income from invertebrate harvesting, as well as experiences, household structure, specific needs and interests related to the activity to organize and transcribe the respondents' answers. Some results from the semi-structured interview provided numerical and binominal data, i.e. “positive” (yes = 1) or “negative” (no = 0); we here performed a nonparametric Multi-Dimensional Scaling ordination (MDS) using the Bray-Curtis similarity measure on fourth root transformed data to analyze patterns in targeted species among male and female harvesters. We further used a one-way analysis of similarities (ANOSIM) to test for differences in targeted species among the sexes and the similarity percentages analysis (SIMPER) was carried out to identify which species contributed most to dissimilarities between males and females. Multivariate analysis was done in PRIMER version 6 software packages.

#### Catch assessments and biological inventories

Permutational multivariate analysis of variance (PERMANOVA) [37] was used to detect possible significant differences in catch data between year and sex, as well as interactions between those. The response variables were i) animal abundance and ii) weight, whereas the independent variables in both analyses were ‘year’ and ‘sex’. A two-way similarity percentage analysis (SIMPER) was used for both abundance and weight data to identify those species that contributed most to dissimilarities between year and sex. The biological inventories included both animal abundance and substrate data; with primary focus on seagrass cover since seagrass meadows are key habitat providers. A permutational analysis of covariance (PERMANCOVA) on two factors (year and site), and with substrate as covariables was done. The site ‘Mabogani’ was removed from the statistical analysis due to the insufficient number of replicates. A two-way SIMPER analysis was carried out to identify those invertebrate species responsible for dissimilarities in abundance within site and between years. Although ‘site’ was initially included in the analysis, the focus of the result and discussion is primarily on temporal differences in the Bay as a whole, and not within specific sites. Multivariate analyses were performed using the software PRIMER 6 plus PERMANOVA. To meet basic statistical assumptions all data was fourth root-transformed. In all analyses, significant levels were considered at α = 0.05.

## Results

### Time spent on collection activities

The semi-structured interview results showed gender disparities in time spent on collection activities; however, no major difference was found between years. In general, women were more restricted by tidal fluctuations and worked during low tide only (3–5 days per week), whereas men worked more frequently on a daily basis during both spring and neap tide. The interviews revealed that this difference was a result of women's limited access to boat transport and a general lack of equipment such as snorkel, flippers and spears. Of all females interviewed less than 60% had access to boat transport. Those that reported to have access to transport commonly asked fishers for boat rides; however, boats were often full and in case of available seats a fee was required. In addition, none of the female respondents had sufficient swimming-skills or knowledge in how to use the equipment mentioned above, but instead used their hands or a wooden stick to collect. In comparison, 98% of the males had access to transport, including both powered and unpowered, and equipment. This allowed them to use two methods; gleaning and breath hold diving to collect, which not only opens up access to both shallow and deep areas but also allows them to be less dependent on tides and other people in their performance of the activity. In addition, the in-depth interviews with both male and female harvesters showed stereotypical gender roles within the household. Men were responsible for bringing income and took most important decisions whereas women took care of household duties such as cooking, cleaning and looking after the children. As a result, the females interviewed experienced little “free time”, which could partly explain differences in time spent on harvesting activities.

### Fishing/collecting grounds and substrate

This typical division of labour not only leads to time constraints but also limits women's mobility and restricts them to areas closer to the household. As a result, they accessed fewer fishing grounds than their male counterparts and the number of good areas with high animal abundance had decreased from 46 in 2005 to 37 in 2010, whereas those of men had increased. Previously, most men fished in deep water only, but due to declining invertebrate abundances in 2010, fishers utilized the shallower areas as well and accessed a total of 84 different sites. Some 11 sites were identified as most popular and used by both male and female harvesters (for site description see Table S1). Based on responses from the semi-structured interviews the popularity of these sites was related to accessibility, animal abundance and substrate. Seagrass meadows followed by sand, mangroves and hard substrate were the most popular habitats among female harvesters whereas male harvesters preferred sandy and rocky bottoms but also seagrasses. The same results were found in 2010; however, the mangrove-area had become increasingly important for females, which was reported to be a result of increasing competition over space among collectors and fewer animals on previously used sites.

### Targeted invertebrate species

**Figure 2:**
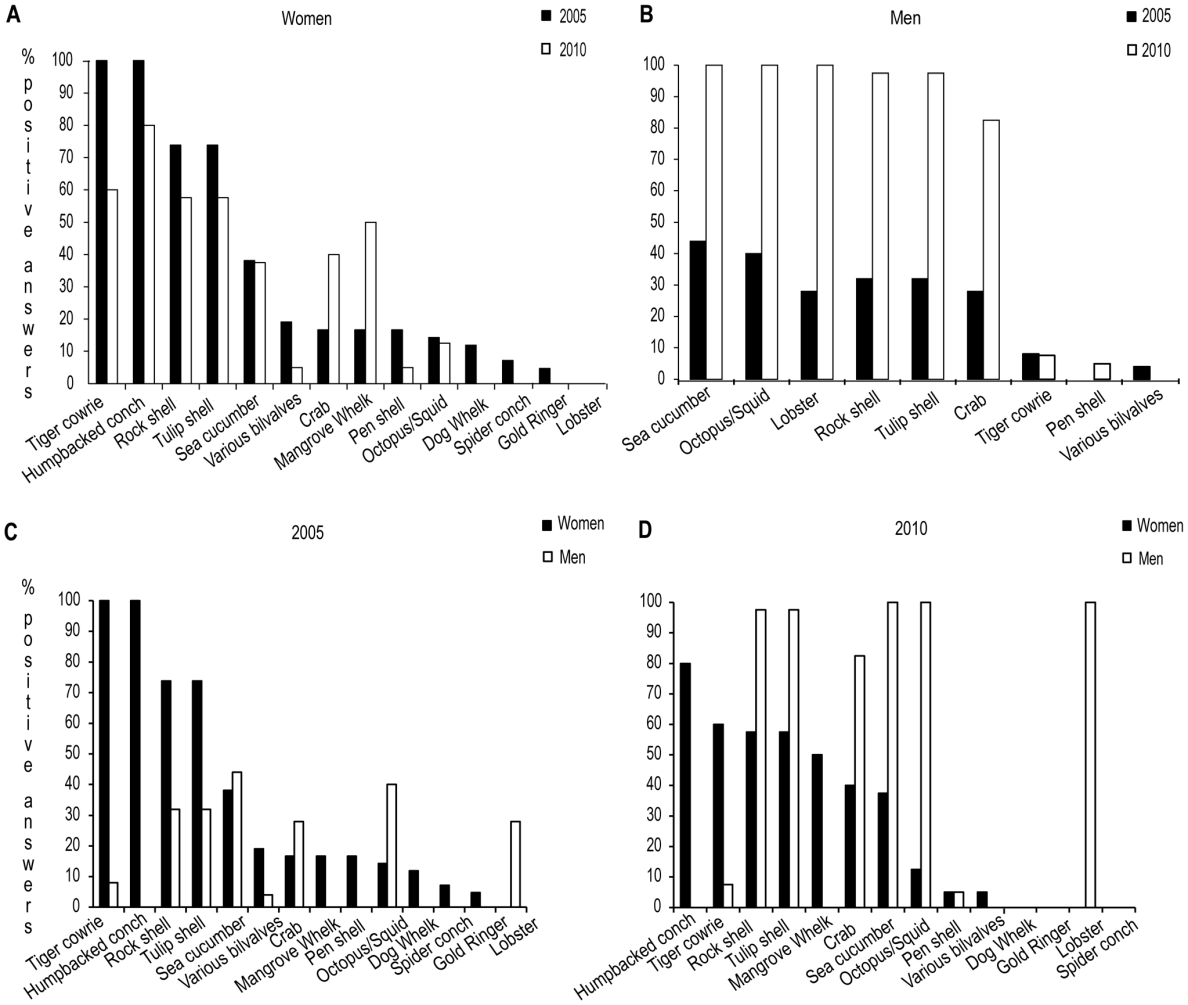
Temoral changes in targeted species *within* gender a) females 2005 (n = 42) and 2010 (n = 40), b) males 2005 (n = 8) and 2010 (n = 40) and *between* gender; c) females (n = 42) and males (n = 8) 2005, d) females (n = 40) and males (n = 40) 2010.

**Figure 3:**
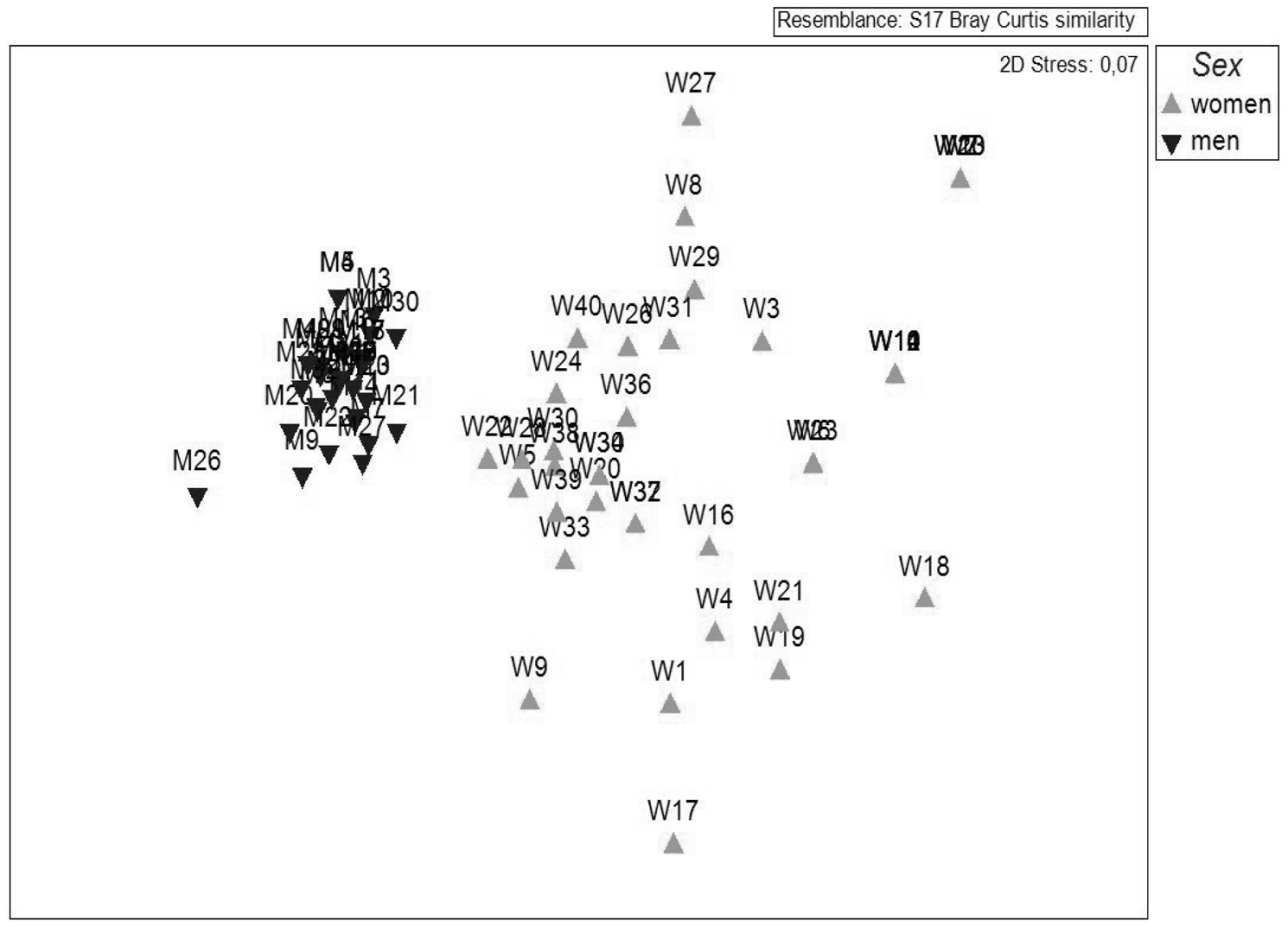
MDS plot illustrating variations in targeted species *between* and *within* the groups of males (n = 40) and females (n = 40) surveyed in 2010. Differences are represented by distance. Data is based on % positive answers.

The semi-structured interview results showed that females typically target a variety of gastropods and bivalves while males instead focus on a few species of high commercial value such as sea cucumber (Holothuroidea spp.), octopus and squid (Cephalopoda spp.), and lobster (Palinura spp.). Targeted species differed somewhat between years; the females surveyed in 2005 mainly harvested the gastropods Tiger cowrie (*Cypraea tigris*, Cypraeidae), Humpbacked conch (*Strombus gibberulus*, Strombidae), Rock shell (*Chicoreus ramosus*, Muricidae) and Tulip shell (*Pleuroploca trapezium*, Fasciolaridae); however, in 2010 the harvest of some of these species had slightly decreased whereas that of Mangrove whelk (*Terebralia palustris*, Potamididae) and crab (Brachyura spp.) had instead increased (Fig. 2a). Both species were typically collected in the mangrove-area. Collection of the gastropod Giant Spider conch (*Lambis truncata*, Strombidae) had completely stopped in 2010. Male harvesters targeted high-value species both years but in 2010 they did not focus on a single species but had become more opportunistic (Fig. 2b). The separation of species according to sex was confirmed by multivariate analysis (ANOSIM global *R* = 0.725; *p*<0.01) (Fig. 2c–d, Fig. 3). The SIMPER analysis further showed that the average dissimilarity between female and male harvesters was 76.59% (Table 1). From this, about 50% was attributed to lobster, eel, octopus/squid, ray and Humpbacked conch.

**Table 1:** Statistical analyses and results of gender-based differences in collected species, size and weight of catches over time, as well as temporal and spatial variations in animal abundance, species richness and substrate between 2005 and 2010.

*Source*	*Method*	*Response*	*Factor*	*df*	*Global R*	*Pseudo F*	*p*
ANOSIM	Interviews	Species	Sex	-	0.725	-	<0.01
PERMANOVA	Catch assessments	Abundance	Year	1		19.84	<0.01
			Sex	1		22.31	<0.01
			Year x sex	1		14.18	<0.01
PERMANOVA	Catch assessments	Weight	Year	1		31.85	<0.01
			Sex	1		11.18	<0.01
PERMANCOVA	Inventories	Abundance	Year	1		7.12	<0.01
			Site	9		16.49	<0.01
		Covariance	Seagrass (year)	1		1.68	0.12
			Seagrass (site)	8		2.98	<0.01
Source	Method	Response	Group	Across groups		Av. sim	Av. diss.
SIMPER	Interviews	Species	Female			45.31	
			Male			83.94	
			Female x Male				76.59
	Catch assessments	Abundance	Female	Year		24.34	
			Male	Year		35.43	
			Female x Male	Year			93.59
SIMPER	Catch assessments	Weight	Female	Year		24.89	
			Male	Year		26.48	
							87.37
			Female x Male	Year			
SIMPER	Inventories	Abundance	2005	Site		49.32	
			2010	Site		40.29	
			2005×2010	Site			83.93

### Invertebrate catch assessments

**Figure 4:**
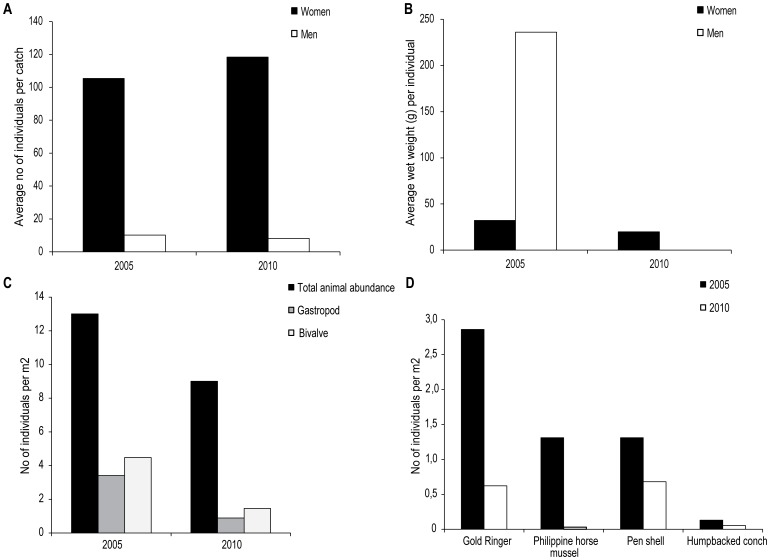
Differences in a) average number of animals per catch between female and male harvesters, b) differences in average wet weight per catch between female and male harvesters, c) differences in total number of invertebrates, gastropods and bivalves per m^2^, and d) differences in total number of Gold Ringer, Philippine horse mussel, Pen shell and Humpbacked conch per m^2^ between 2005 and 2010.

Catches by female harvesters showed a small increase in numbers of collected animals between the years; from an average number of 105.4 individuals per catch in 2005 to 118.4 in 2010. In 2005 their catches were dominated by Humpbacked conch (*S. gibberulus*), Philippine horse mussel (*Modiolus philippinarum*, Mytilidae) and Tiger cowrie (*C. tigris*). However, in 2010 catches mainly contained Gold ringer (*Cypraea annulus*, Cypraediae), Mangrove Whelk (*T. palustris*) and Humpbacked conch (all species caught are shown in Table 2). Catches by male harvesters commonly contained fewer but larger species; for example, in 2005 their catches were dominated by Rock shell (*C. ramosus*), Pen shell (*Atrina vexillum*, Pinnidae), Tulip shell (*P. trapezium*) and Giant Spider conch (*L. truncata*), whereas in 2010 octopus and crab accounted for large parts of the catch. Unlike the catches by females the average number of individuals per catch had decreased from 10.2 in 2005 to 8.2 in 2010 (Table 3). These temporal and gender-based differences in catch data were demonstrated by the PERMANOVA; significant differences in catch abundance between years (*F* = 19.84; *p*<0.01) and sex (*F* = 22.31; *p*<0.01) and an interaction between those (*F* = 14.18; *p*<0.01) was found (Table 1, Fig. 4a). The SIMPER analysis showed that the average dissimilarity in animal abundance of catches between the sexes across all year groups (2005, 2010) (93.59%) (Table 1), was mainly attributed to Mangrove Whelk (14.39%), Octopus (11.06%) Tiger cowrie (10.54%), Humpbacked conch (8.15%) and Rock shell (5.65%).

**Table 2:** Summary of women's catches for both 2005 and 2010 in Chwaka village, Zanzibar, Tanzania.

*Species/Taxonomic groups*	*Ind. per catch 2005*	*Ind. per catch 2010*	*Wet weight per catch (g) 2005*	*Wet weight per catch (g) 2010*	*Wet weight per ind. (g) 2005*	*Wet weight per ind. (g) 2010*
*Strombus gibberulus*	46.6	21.9	547.0	228.8	11.7	10.5
*Modiolus philippinarum*	19.4	0.1	185.0	0.6	9.5	7.4
*Cypraea tigris*	13.0	2.5	2004.0	321.2	154.2	127.5
Neritiidae	7.9	11.5	44.0	33.4	5.6	2.9
Chitonidae	4.4	3.3	36.0	24.1	8.2	7.3
Other gastropods	2.9	18.1	63.0	61.1	21.7	3.4
*Cypraea annulus*	2.3	30.5	6.0	74.6	2.6	2.4
Nassariidae	2.0	2.0	4.0	4.8	2.0	2.4
*Terebralia palustris*	1.6	22.7	119.0	1442.8	74.4	63.7
Other bivalves	1.6	0.3	42.0	5.8	26.3	21.5
*Volema pyrym*	1.5	2.9	27.0	65.6	18.0	22.5
*Pinna muricata*	0.6	<0.1	57.0	0.4	95.0	10.0
*Chicoreus ramosus*	0.6	0.2	107.0	38.1	178.3	158.7
*Atrina vexillum*	0.3	<0.1	80.0	3.7	266.7	186.5
*Pleuroploca trapezium*	0.3	0.2	37.0	21.8	123.3	120.9
Starfish	0.1	-	4.0	-	40.0	-
Brittlestar	-	1.0	-	0.1	-	0.1
Sea cucumber	0.1	0.1	21.0	0.9	210.0	9.0
Octopus	-	1.0	-	9.3	-	9.3
Squid	0.1	-	1.0	-	10.0	-
Crab	0.1	0.1	8.0	12.9	80.0	129.0
*Total all species*	*105.4*	*118.4*	*3392.0*	*2349.9*	*32.2*	*19.9*

**Table 3:** Summary of men's catches for both 2005 and 2010 in Chwaka village, Zanzibar, Tanzania.

*Species/Taxonomic group*	*Ind. per catch 2005*	*Ind. per catch 2010*	*Wet weight per catch (g) 2005*	*Wet weight per ind. (g) 2005*
*Chicoreus ramosus*	3.0	0.5	675.0	225.0
*Atrina vexillum*	1.9	-	890.0	468.4
*Pleuroploca trapezium*	1.9	0.1	240.0	126.3
*Lambis truncata*	1.4	-	191.0	136.4
*Portunus pelagicus (crab)*	0.8	1.2	201.0	287.1
*Panulirus ornatus (lobster)*	0.8	0.1	12.0	120.0
*Cypraea tigris*	0.7	0.7	124.0	177.1
*Pinctada margaritifera (oyster)*	0.1	-	12.0	120.0
Conidae	0.1	-	8.0	80.0
Cypraea	0.1	-	6.0	60.0
*Acrosterigma rubicundum*	0.1	-	3.0	30.0
*Polinices mamilla*	0.1	-	1.0	10.0
Octopus	-	4.3	-	-
Squid	-	0.1	-	-
Sea cucumber	-	0.8	-	-
Eel	-	0.5	-	-
*Total all species*	*10.2*	*8.2*	*2363.0*	*236.0*

Whereas the number of individuals per catch had increased for the female harvesters the average wet weight of animals had decreased from 32.2 g in 2005 to 19.9 g per individual in 2010 (Table 2). For males, data is only available for 2005; however, the average weight per individual was 236 g, which is considerably higher than for females (Table 3). Commercially valuable species of sea cucumber and lobster were rarely found in either the catches from females or males in 2005 and 2010. The Giant Spider conch, which was frequently collected in 2005, was not found in any of the catches analyzed in 2010. The PERMANOVA showed significant differences for weight data between years (*F* = 31.85; *p*<0.01) and sex (*F* = 11.18; *p*<0.01) (Table 1, Fig. 4b). The SIMPER analysis showed that the average dissimilarity in weight of individuals between the sexes across all year groups (2005, 2010) (87.37%) was mainly attributed to Tiger cowrie (18.37%), Rock shell (12.53%), Pen shell (11.82%), Humpbacked conch (9.07%) and Tulip shell (8.54%).

### Resource status and changes (2005–2010)

The inventories of invertebrate abundance on popular collecting grounds showed a total number of 67 different species/taxonomic groups both years (complete list of inventories is found in Table S2). However, in 2005 a total number of 2795 animals were found, giving an average number of 13 per m^2^, whereas in 2010 a total number of 2015 animals, giving an average number of 9 animals per m^2^, were found. The average number of gastropods and bivalves had sharply decreased from 716 to 206 (71%) and 944 to 339 (64%), respectively (Fig. 4c). The PERMANCOVA showed significant differences in animal abundance between years (*F* = 7.12; *p*<0.01) and within site (*F* = 16.49; *p*<0.01) (Table 1). Significant covariance was also found for sites (*F* = 2.98; *p*<0.01) but there was no significant relationship between changes in animal abundance and seagrass cover over years (*F* = 1.68; *p* = 0.12) (Table 1). The SIMPER analysis showed that 25 species contributed to the average dissimilarity (83.93%) in animal abundance between year 2005 and 2010 (across all site groups). From this about 28% was attributed to two species of non-harvested sea urchins (*Diadema savignyi*, Diadematidae and *Echinometra mathaei*, Echinometridae) with 13.54% and 13.46%, respectively. However, other species that contributed was the commonly collected Gold Ringer (8.78%), Pen Shell (8.21%), Philippine horse mussel (4.36%) and Humpbacked conch (2.56%), which had all decreased in a five-year period (Fig. 4d). Similar to the catch analysis, the Giant Spider conch and economically valuable species of sea cucumber and lobster were not found during inventories. In addition, the semi-structured interviews from 2010 show that 80% of the males and 95% of the females perceived a decrease in collected animals.

### Livelihood contributions of the invertebrate fishery

According to the results from semi-structured interviews, the use of invertebrates differed depending on needs at the time and between species. The meat from smaller and less economically valuable invertebrates was consumed at home whereas species of higher economic value and shells were sold (Table S3). In 2005, 88% of the males and 88% of the females considered invertebrates as “very important” for income, whereas only 12.5% of the males and 4% of the females considered it as “very important” for subsistence. In 2010, 73% of the males and 85% of the females still considered the activity as “very important” for income while the importance for subsistence had increased to 40% for males and 12.5% for females. This was reported as a result of declines in economically valuable species but also related to high prices of finfish, which subsequently had augmented the importance of invertebrates for home consumption. When looking at specific species, commercially valuable species of sea cucumbers and the gastropods Tiger cowrie, Rock shell, Tulip shell, as well as the operculum from the two latter, were in 2005 exclusively sold, generally by men and via middlemen from Zanzibar Town. In 2010, sea cucumbers were still exclusively sold via middlemen, whereas Tiger cowrie, Rock shell and Tulip shell had become increasingly important as food. The in-depth interview results showed that differences in market access and diverse customers were strongly related to men's greater mobility, but also to “free time” that could be spent on establishing important contacts. These factors resulted in income differentials; the male respondents reported a daily income more than five times that of the females. Despite the fact that the income of female harvesters had increased from about 600 (min/max 1–2 000) TZS (0.6 USD) (1 000 TZS was equivalent to about 1 US dollar at the time for the study) in 2005 to 986 (min/max 0–10 000) TZS (about 1 USD) in 2010, their daily income was often below the extreme poverty line of 1.25 US dollar per day. Due to low incomes about 57% of the females interviewed in 2005 and 60% of the females interviewed in 2010 were involved in additional activities such as seaweed farming and petty business related to food items sale. Although the income of male harvesters had decreased from about 10 000 (min/max 0–20 000) TZS (10 USD) in 2005 to 5 600 (min/max 0–57 000) TZS (5.6 USD) in 2010, only 10% were involved in additional activities such as tourism-related work or driving local buses.

### General needs and interests among harvesters

The in-depth interviews encouraged respondents to develop their own ideas and thoughts on particular issues, needs and interests associated with this activity. Both male and female respondents reported that invertebrate harvesting is one of the few livelihood options available and despite the low income generated and declining catches they still continue to collect. For example, starting up a new business, such as a small shop, requires financial, social (contacts) and human (training) capital; something that most of them, and particularly women, stated not to have. To improve current conditions all women interviewed requested greater access to financial capital, social capital, such as a broader contact network, that could open up access to a bigger market. They also highlighted the need for physical capital including boat transport and protective footwear. Men, who to a greater extent had access to transport and markets, instead requested knowledge and training in how to protect resources from overexploitation. One key issue brought up during all in-depth interviews was the lack of a formal organization, which can work as an arena for discussion between collectors, fisheries managers and policy-makers.

## Discussion

### Temporal changes in resource status

One limitation of the presented study is the lack of baseline data against which our data can be compared. Ideally, data should be sampled yearly to study time trends but such data is lacking in many developing countries and Chwaka Bay is no exception. Another limitation is the lack of environmental measurements, such as seawater temperatures and quality, which together with natural variations could be potential drivers behind change. However, the findings from this case illustrate how unregulated harvesting of invertebrates may have negative ecological effects in a mid-term perspective; over a period of five years significant changes have occurred. Similar to recent studies on invertebrate fisheries [9], [38], our study challenges the old view of many invertebrate species as resilient to over-exploitation [3], [39]. Significant declines were particularly evident for prime target organisms such as Gold ringer (*C. annulus*), Pen shell (*P. muricata*), Humpbacked conch (*S. gibberulus*), Philippine horse mussel (*M. philippinarum*), and Tiger cowrie (*C. tigris*). Also, the catch analysis revealed a weight reduction for many of the collected species; especially Tiger cowrie and Humpbacked conch had been reduced both in size and number. In addition, invertebrates frequently collected by males in 2005, such as the Giant Spider conch (*L. truncata*), Rock shell (*C. ramosus*), Tulip shell (*P. trapezium*) and high-valued species of lobster and sea cucumber, were not found in the inventories and rarely observed in catches in 2010. This is coherent with a study by Jamieson [3]; particularly species of i) relatively large size, ii) that occur in shallow water close to human settlement, iii) have an exceptional market demand, and iv) a relatively long life span are vulnerable to over-exploitation. Sea cucumbers and many of the targeted gastropods (e.g. Humpbacked conch, Rock shell and Tiger cowrie) fall under this category and may thus be subject to high collection pressure due to their high value. A decline in prime target invertebrates corresponds to previous findings from Chwaka Bay [18], [26], [40]–[42], mainland Tanzania [43]–[45] and other countries such as Chile [46]. Another well-known example is that of the white abalone in California and Mexico that shows how harvesting practices can cause serious declines, which in this case resulted in the white abalone being the first marine invertebrate to be proposed for the US endangered species list [47]. In the high-valued sea cucumber fishery [7], [40], decreasing catch trends have been directly linked to animal population declines due to easiness in harvesting; however, declines in animal abundance may also be related to natural drivers such as unsuccessful recruitment due to climate [2] and/or habitat disturbance. Habitat wise, it is well known that healthy seagrass meadows sustain higher species richness than unvegetated habitats [19]–[20]. Although studies from Chwaka Bay (e.g. [48]), indicate a seagrass decline for specific areas there was no significant covariance between changes in animal abundance and seagrass cover over year. Thus, changes in habitat structure, at least in this case, seem not to be the main driver behind change. In addition, data was sampled in spring/summer both years, which should minimize the risk of seasonal effects in invertebrate abundance. However, there may still be other potential drivers such as changes in seawater temperature, quality or natural variations in population recruitment that were not investigated in this study. To understand 1) what drives invertebrate declines, 2) how different drivers can act in conjunction with each other, 3) the functional role of different invertebrates and 4) the possible consequences for ecosystem health by such removal, research over time is certainly needed. With a few exceptions (e.g. [5]) the ecological consequences of many invertebrate fisheries are largely unknown and unassessed [2]. For example, the links between invertebrates and surrounding ecosystems such as seagrasses [49], as well as the ecological implications of diminishing specific resource stocks [50], require further attention. Although this study does not analyse all the potential set of factors affecting invertebrate populations, the results still point towards intense harvesting as an important driver behind invertebrate declines. This paper should also be a valuable contribution and serve a function as baseline for future studies.

### Livelihoods and gender

Invertebrate harvesting is one of a few livelihood options available to the population in Chwaka village and in only five years at least twice as many households have entered the fishery. Despite low profits the contribution of invertebrate harvesting to income of both individual collectors and the household as a whole is therefore important to consider. However, with declining finfish fisheries invertebrates may also come to play a more important role as food, which has been observed in other parts of Zanzibar such as Nungwi [45]; the Comoros islands [4] and Mozambique [51]. In the light of this, it is essential to analyze the potential impact of declining invertebrate resources on people's livelihoods and how livelihood strategies and vulnerability in the context of changing social-ecological conditions may differ according to gender. The results show that a decline in economically valuable species have forced males to start targeting species of lower value in shallow areas previously utilized by females only, which could result in female harvesters being displaced from good fishing areas and thus outcompeted in their pursue of invertebrate resources. This is to some extent already observed; male harvesters were more mobile and able to shift fishing grounds whereas females generally could not. These gender inequalities are deeply rooted within the society, and women's reproductive role is typically valued higher than their productive. The findings also show that limited mobility and lack of “free time” reduce their chances to accumulate financial, physical, social and human capital needed to cope with changes in the environment. One limitation regarding the interviews is the skewed number of male respondents between years; however, the gender-based differences found in this study are not unique for Zanzibar but seen in the fisheries sector all over the world such as sub-Saharan Africa [52]–[53], India, Malaysia, Cambodia [54], and Brazil [55]. Subsequently, in line with the livelihoods framework [15] women's livelihood assets differ from that of men, which may have negative impacts on livelihood strategies, not only for the individual but for whole households, and further lead to increased vulnerability in the context of declining invertebrate resources, price shocks, market changes and so on.

### Conservation and management of fisheries resources

Our results show trends in invertebrate fisheries that are far from optimistic. They also demonstrate how invertebrate harvesting is, similar to other types of fisheries, a constant tradeoff between the ecological and the socio-economic system [56]. However, despite the important role invertebrates play for both income generation and subsistence in coastal communities such as Chwaka Bay, there is currently no formal management regime targeting invertebrate fisheries specifically. For example, the legal framework guiding fisheries policy and management such as the Fisheries Acts [57]–[59] interpret invertebrates generically as “fish”, which refers to all forms of aquatic or amphibious life. Subsequently, there is a tendency to focus on finfish fisheries, including management measures, gear restrictions and size of fish caught. This is the case also with the village fisheries committees, and although invertebrate collectors are welcome to join, most of them do not. However, the Institute of Marine Science, together with the Western Indian Ocean Marine Science Association, have made some attempts to manage declining invertebrate resources while at the same involving local harvesters. Four no-take zones have been established in Menai Bay, located on the south-western tip of Zanzibar; by-laws and management plans have further been developed and signed by both village heads and the District Fisheries Commissioner. However, despite these efforts only one of the four no-take zones showed an increase in animal abundance within a three year period (2006–2008), which was assumed to be a result of insufficient sizes of established zones and improper site and habitat selection, but also poaching by neighbouring villages [44]. In addition, there has been no change in fisheries policies either at Zanzibar or at national level regarding invertebrates. However, other places such as Chile have been more successful in managing invertebrate resources [5]. Yet, our study illustrates the importance of long-term studies to understand the functional role of targeted invertebrates, life cycles, and how they respond to exploitation, but also to consider social factors of the specific location before implementing any form of management. In Zanzibar where knowledge about the ecological function of invertebrates is generally scarce, monitoring absent, and harvesters' needs and interests typically overlooked, it is high time to act. Gathering comprehensive information on the functional role of species and ecological processes could take time, but was set as the highest priority in a study on experts' opinions for intertidal zone management in the WIO [60]. However, some actions such as improved monitoring could be a lot faster to implement. There are monitoring agents (*Bwana Dikos*) employed by the Government that mainly regulate finfish fisheries [25], and one idea is to institute “Invertebrate *Bwana Dikos*” [26]. This would not only improve monitoring but also provide new job opportunities in the villages. A case from Australia showed how local fisheries contributed greatly to data collection [61]. Another example demonstrates the positive effects of women's participation in forest conservation in India; here local NGO's played a major role by putting pressure on state governments to establish committees inclusive of women, which resulted in an increased participation and bargaining power essential for bringing about change [62]. In addition, the Government should establish formal organizations similar to the fisheries committee, which could act as a platform for collecting gender-disaggregated statistics about number of harvesters, sex, collected species, and economic structures of the fishery. Such data is essential in order to make robust analyses [10] but could also help making women's contribution to the fishery, and household economy and subsistence supply, more visible to policy-makers and managers. In conclusion, to make policy and management advances in sustainable invertebrate fisheries, a multifaceted approach is needed considering knowledge about environment – species interactions, the social context of a specific location and the changes observed over time. It should further include gender as an integral part of any analysis and management intervention to accommodate the roles and interests of both men and women, which is essential for sustainable resource utilization and development.

## Supporting Information

Table S1 **Site description of the 11 most popular fishing/collecting grounds.**
(DOCX)

Table S2 **Total number of species inventoried at the 11 most popular fishing/collecting grounds in Chwaka Bay 2005 and 2010.**
(DOCX)

Table S3 **Usage, actual price and inflation-adjusted price for common invertebrate species.** Price is given per piece or kg and in Tanzanian shilling (TZS).
(DOCX)

Text S1 **Interview forms.**
(DOCX)
