# Supplementary material for: Towards Improved Management of Tropical Invertebrate Fisheries: Including Time Series and Gender
Source: PLoS One. 2014 Mar 10;9(3):e91161. doi: 10.1371/journal.pone.0091161 (PMC3948745; doi:10.1371/journal.pone.0091161)
Supplement: Table S1 — Site description of the 11 most popular fishing/collecting grounds. (DOCX) [file pone.0091161.s001.docx]

**Table S1** Site description of the 11 most popular fishing/collecting grounds.

| *Fishing/collecting grounds* | *General description* |
| --- | --- |
| Bimbi | Dominated by packed sand covering the underlying limestone. No algae or seagrass present but some patches of coral rubble. High abundance of starfish and sea urchins. Also bivalves of the family Mytilidae occurred in high numbers. |
| Charawe | Mixed substrate of seagrass, sand, some halimeda, other algae and sponge. Several species of seagrasses were observed. Relatively few invertebrates; however, the bulky sea hare *Dolabella auricularia* was rather abundant. |
| Chumani | Mixed substrate of seagrasses, sand, halimeda, algae and sponge. High abundance of *E. mathaei.* Another relatively common organism was the shallow-living nudibranch *Melibe pilosa*. Some specimens of Tiger cowrie (*C. tigris*) were observed. |
| Haibari | Mixed vegetation of sand, seagrass, halimeda and other algae with a high level of patchiness. Several seagrass farms were situated in the area. The detritus-feeding sea cucumber *Synapta maculata* was commonly observed. |
| Juja | Dominated by sand with some seagrass, Thalassia spp., and patches of halimeda. High abundances of *E. mathaei* and the burying Pen shell, *P. muricata,* were observed. |
| Mabogani | Substrate dominated by sand with some patches of halimeda and seagrass. Very few organisms found except for a few Rock shells (*S. gibberulus*). |
| Mreno | Dominated by halimeda and with some sand patches. Some seagrass present. High number *of E. mathei* |
| Mto Mwembamba | Mixed substrate of seagrass, sand, patches of halimeda and relatively high coverage of sponge. High level of patchiness. High abundances of *E. mathaei* and relatively high numbers of cowries were found. |
| Mwachange | Dominated by sand with high abundances of starfish and sea urchins. Also bivalves of the family Mytilidae occurred in high numbers. |
| Pondoni | Mixed substrate of seagrass, sand, halimeda and other algae. Some sponge. *E. mathaei* was the dominating species, relatively few other species found |
| Shahaji | Substrate constituted of a mix of sand and seagrass. Many seaweed farms in the area. |
