# Supplementary material for: Towards Improved Management of Tropical Invertebrate Fisheries: Including Time Series and Gender
Source: PLoS One. 2014 Mar 10;9(3):e91161. doi: 10.1371/journal.pone.0091161 (PMC3948745; doi:10.1371/journal.pone.0091161)
Supplement: Table S2 — Total number of species inventoried at the 11 most popular fishing/collecting grounds in Chwaka Bay 2005 and 2010. (DOCX) [file pone.0091161.s002.docx]

**Table S2** Total number of species inventoried at the 11 most popular fishing/collecting grounds in Chwaka Bay 2005 and 2010.

| *Class* | *Species* | *Total 2005* | *Total 2010* | *Average 2005* | *Average 2010* | *Average per m^2^ 2005* | *Average per m^2^ 2010* |
| --- | --- | --- | --- | --- | --- | --- | --- |
| Flatworm | Pseudocerotidae | 0.00 | 0.00 | 0.00 | 0.00 | 0.00 | 0.00 |
|  | Flatworm unid. | 0.00 | 3.00 | 0.00 | 0.27 | 0.00 | 0.01 |
| Gastropod | Trochidae | 6.00 | 15.00 | 0.55 | 1.35 | 0.03 | 0.06 |
|  | *Turbo marmoratus* | 1.00 | 0.00 | 0.09 | 0.00 | 0.01 | 0.00 |
|  | Cerithiidae | 2.00 | 7.00 | 0.18 | 0.64 | 0.01 | 0.03 |
|  | *Strombus gibberulus* | 28.00 | 12.00 | 2.55 | 1.09 | 0.13 | 0.05 |
|  | Strombidae | 0.00 | 1.00 | 0.00 | 0.09 | 0.00 | 0.00 |
|  | *Polinices mammilla* | 0.00 | 1.00 | 0.00 | 0.09 | 0.00 | 0.00 |
|  | Natacidae | 2.00 | 0.00 | 0.18 | 0.00 | 0.01 | 0.00 |
|  | *Cypraea annulus* | 603.00 | 144.00 | 54.82 | 13.10 | 2.86 | 0.62 |
|  | *Cypraea arabica* | 0.00 | 1.00 | 0.00 | 0.09 | 0.00 | 0.00 |
|  | *Cypraea carneola* | 0.00 | 1.00 | 0.00 | 0.09 | 0.00 | 0.00 |
|  | *Cypraea helvola* | 1.00 | 2.00 | 0.09 | 0.18 | 0.01 | 0.01 |
|  | *Cypraea moneta* | 3.00 | 4.00 | 0.27 | 0.36 | 0.01 | 0.02 |
|  | *Cypraea tigris* | 4.00 | 3.00 | 0.36 | 0.27 | 0.02 | 0.01 |
|  | *Cypraea vitellus* | 0.00 | 2.00 | 0.00 | 0.17 | 0.00 | 0.01 |
|  | Ovulidae | 0.00 | 1.00 | 0.00 | 0.09 | 0.00 | 0.00 |
|  | *Cymatium muricinum* | 0.00 | 4.00 | 0.00 | 0.36 | 0.00 | 0.02 |
|  | Ranellidae | 4.00 | 1.00 | 0.36 | 0.09 | 0.02 | 0.00 |
|  | *Chicoreus ramosus* | 2.00 | 0.00 | 0.18 | 0.00 | 0.01 | 0.00 |
|  | *Nassarius cornatus* | 1.00 | 1.00 | 0.09 | 1.00 | 0.01 | 0.01 |
|  | Fasciolaridae | 0.00 | 1.00 | 0.00 | 0.09 | 0.00 | 0.00 |
|  | *Vasum rhinoceros* | 1.00 | 1.00 | 0.09 | 0.09 | 0.01 | 0.00 |
|  | Conidae | 8.00 | 3.00 | 0.73 | 0.27 | 0.03 | 0.01 |
|  | Gastropod unid. | 50.00 | 1.00 | 4.55 | 0.09 | 0.24 | 0.00 |
| Sea slug | *Bulla ampulla* | 2.00 | 10.00 | 0.18 | 0.09 | 0.01 | 0.04 |
|  | *Dolabella auricularia* | 10.00 | 25.00 | 0.91 | 2.27 | 0.05 | 0.11 |
|  | *Berthellina citrina* | 0.00 | 29.00 | 0.00 | 2.64 | 0.00 | 0.13 |
|  | *Hexabranchus marginatus* | 0.00 | 1.00 | 0.00 | 0.09 | 0.00 | 0.00 |
|  | *Melibe pilosa* | 0.00 | 9.00 | 0.00 | 0.81 | 0.00 | 0.04 |
|  | Nudibranch unid. | 3.00 | 8.00 | 0.27 | 0.73 | 0.01 | 0.03 |
| Bivalve | *Anadaria antiquata* | 6.00 | 2.00 | 0.55 | 0.18 | 0.02 | 0.01 |
|  | Glycymerididae | 0.00 | 4.00 | 0.00 | 0.36 | 0.00 | 0.02 |
|  | *Modiolus philippinarum* | 278.00 | 7.00 | 25.27 | 0.64 | 1.31 | 0.03 |
|  | *Septifer bilocularis* | 1.00 | 0.00 | 0.09 | 0.00 | 0.01 | 0.00 |
|  | Mytilidae | 1.00 | 69.00 | 0.09 | 6.27 | 0.01 | 0.30 |
|  | *Pinna muricata* | 277.00 | 158.00 | 25.18 | 14.40 | 1.31 | 0.68 |
|  | *Isognomon ephippium* | 0.00 | 1.00 | 0.00 | 0.09 | 0.00 | 0.00 |
|  | Lucinidae | 321.00 | 0.00 | 29.18 | 0.00 | 1.52 | 0.00 |
|  | Tridacna | 1.00 | 3.00 | 0.09 | 0.27 | 0.01 | 0.01 |
|  | *Acrosterigma rubicundum* | 10.00 | 4.00 | 0.91 | 0.36 | 0.05 | 0.02 |
|  | *Lunulicardia auricula* | 2.00 | 0.00 | 0.18 | 0.00 | 0.01 | 0.00 |
|  | *Cardita variegata* | 0.00 | 1.00 | 0.00 | 0.09 | 0.00 | 0.00 |
|  | *Gafrarium pectinatum* | 29.00 | 13.00 | 2.64 | 1.18 | 0.14 | 0.06 |
|  | *Pitar hebraea* | 10.00 | 0.00 | 0.91 | 0.00 | 0.05 | 0.00 |
|  | Veneridae | 8.00 | 77.00 | 0.73 | 7.00 | 0.04 | 0.33 |
| Sea urchin | *Diadema savignyi* | 1.00 | 1.00 | 0.09 | 0.18 | 0.01 | 0.01 |
|  | *Tripneustes gratilla* | 18.00 | 13.00 | 1.64 | 1.18 | 0.09 | 0.06 |
|  | *Echinometra mathaei* | 502.00 | 494.00 | 45.64 | 45.00 | 2.38 | 2.13 |
|  | Giant sea urchin | 1.00 | 26.00 | 0.09 | 2.36 | 0.01 | 0.11 |
| Sea cucumber | *Synapta maculata* | 3.00 | 11.00 | 0.27 | 1.00 | 0.01 | 0.05 |
|  | Sea cucumber unid. | 10.00 | 24.00 | 0.91 | 2.18 | 0.04 | 0.10 |
| Starfish | *Culcita schmideliana* | 0.00 | 1.00 | 0.00 | 0.09 | 0.00 | 0.00 |
|  | Pentaceraster | 4.00 | 3.00 | 0.36 | 0.27 | 0.02 | 0.01 |
|  | *Proteaster lincki* | 2.00 | 0.00 | 0.18 | 0.00 | 0.01 | 0.00 |
|  | *Leiacester leichi* | 1.00 | 0.00 | 0.09 | 0.00 | 0.01 | 0.00 |
|  | Ophidiasteridae | 0.00 | 28.00 | 0.00 | 2.54 | 0.00 | 0.12 |
|  | Starfish unid. | 5.00 | 0.00 | 0.45 | 0.00 | 0.02 | 0.00 |
| Brittlestar | Ophiurida | 553.00 | 745.00 | 50.27 | 67.70 | 2.62 | 3.21 |
| Crab | *Calappa hepatica* | 3.00 | 2.00 | 0.27 | 0.18 | 0.01 | 0.01 |
|  | Calappidae | 0.00 | 3.00 | 0.00 | 0.27 | 0.00 | 0.01 |
|  | *Portunus pelagicus* | 3.00 | 0.00 | 0.27 | 0.00 | 0.01 | 0.00 |
|  | Crab unid. | 14.00 | 10.00 | 1.27 | 0.91 | 0.07 | 0.04 |
| Hermit crab | Hermit crabs unid. | 0.00 | 22.00 | 0.00 | 2.00 | 0.00 | 0.09 |
| Sea squirt | Sea squirts unid. | 0.00 | 2.00 | 0.00 | 0.18 | 0.00 | 0.01 |
|  | *Total for all sites* | *2795* | *2015* | *254* | *183* | *13* | *9* |
