# Supplementary material for: Towards Improved Management of Tropical Invertebrate Fisheries: Including Time Series and Gender
Source: PLoS One. 2014 Mar 10;9(3):e91161. doi: 10.1371/journal.pone.0091161 (PMC3948745; doi:10.1371/journal.pone.0091161)
Supplement: Table S3 — Usage, actual price and inflation-adjusted price for common invertebrate species. Price is given per piece or kg and in Tanzanian shilling (TZS). (DOCX) [file pone.0091161.s003.docx]

**Table S3** Usage, actual price and inflation adjusted price for common invertebrate species. Price obtained from monitoring agents and middlemen is given per piece or kg.

| *Species/Taxonomic group* | *Food* | *Income* | *Local price 2005 (TZS)* | *Local price 2013(TZS)* | *Market price 2005(TZS)* | *Market price 2013(TZS)* |
| --- | --- | --- | --- | --- | --- | --- |
| *Cypraea tigris/* Tiger cowrie | Yes | Yes | 30 (6.8) | 40 (4.4) | 40 (9.1) | 1000 - 2000/shell (111.1 - 222.2) |
| *Strombus gibberulus/* Humpbacked conch | Yes | Yes | 2 - 5 (0.5 - 1.1) | - | - | - |
| *Pleuroploca trapezium/* Tulip shell | Yes | Yes | 10 (2.3) | - | 20 - 40/opercula (4.5 - 9.1) 10/shell (2.3) | 75 000/kg opercula (8333.3) |
| *Chicoreus ramosus/* Rock shell | Yes | Yes | 10 (2.3) | - | 20 - 40/opercula (4.5 - 9.1) 10/shell (2.3) | 75 000 kg/opercula (8333.3) |
| Holothuroidea spp/ Sea cucumber | No | Yes | - | - | 200 - 6000 (45.5 - 1363.6) |  |
| *Terebralia palustris/* Mangrove Whelk | Yes | Yes | - | - | - | - |
| *Pinna muricata/Atrina vexillum/* Pen shell | Yes | Yes | 3 - 4 (0.7 - 0.9) | - | - | - |
| *Lambis truncata/* Giant Spider conch | Yes | Yes | - | - | - | - |
| Brachuyra spp./ Crabs | Yes | Yes | - | - |  |  |
| Nassariidae/ Dog Whelk | Yes | No | - | - | - | - |
| Cephalopoda spp./ Octopus/Squid | Yes | Yes | 500 - 700 (113.6 - 159.1) | 3000 - 4000/kg (333.3 -444.4/kg) | - | - |
| Ostreidae/ Oyster | Yes | Yes | - | - | - | - |
| *Cypraea annulus/* Gold Ringer | Yes | Yes | 1(0.2) | 10 (2.5) | 1/shell  (0.2) | - |
| Ovulidae/ Egg shells | Yes | Yes | - | - | - | - |
