# Supplementary material for: Towards Improved Management of Tropical Invertebrate Fisheries: Including Time Series and Gender
Source: PLoS One. 2014 Mar 10;9(3):e91161. doi: 10.1371/journal.pone.0091161 (PMC3948745; doi:10.1371/journal.pone.0091161)
Supplement: Text S1 — Interview forms. (DOCX) [file pone.0091161.s004.docx]

**Text S1** Interview forms

**Personal description**

Name

Are you from Chwaka? If no, from where?

Education:

No education Quran Primary school

Secondary school Other higher

Marital status:

How many children do you have?

Boys................... Girls………………..

1. Do you collect invertebrates?
2. Is it your main job?

If no, what is your main job?

1. How often do you collect invertebrates during one spring tide?
2. How often do you collect invertebrates during neap tide?
3. Do you collect invertebrates on any other times? If yes, when?
4. How important is invertebrate collection/spear fishing for food?
5. How important is invertebrate collection/spear fishing for money?
6. Does the number of animals vary depending on the season? Eexplain
7. When do you catch the highest numbers of animals?
8. Does the time you spend collecting invertebrates/spear fishing animals

change depending on the season? Explain

1. Does the size of your catch change depending on the season? Explain
2. Does the price you sell your catch change depending on the season? Explain
3. In what area do you most often go to collect invertebrates? What types of vegetation/substrate are present where the following animals are most often found?
4. Which substrate do you prefer and why?
5. Why are these places good? Explain.
6. What type of substrate and vegetation are usually present at a good site?
7. Anything else that is important for a good place for collection/spear fishing?
8. Has the vegetation or substrate changed during the past 5 years in any of these sites?
9. Has the number of animals changed?
10. Has the size of your animals changed?
11. Has the number of people collecting invertebrates/spear fishing changed?
12. Is invertebrate collection/spear fishing a good job? Why or why not?
13. Which animals do you collect most of? What do you use them for? If sold, what is the price and to whom do you sell it?
14. Other animals collected
15. What is your weekly income?
16. Are there any animals you would like to catch more of?
17. What is the food value for the animals you get?
18. What is the abundance of these animals?
19. What would you do if you could not collect invertebrates?
20. Why do you want to do that?
21. What would you do if you could not do your main job?
22. Is there any type of job you would like to learn how to do and please explain why?
23. Is there anything that is stopping you from learning that job (skills, money, time, friends, family etc)? Please specify who and why.

**In depth questions**

- Could you tell me about your work as an invertebrate collector? How do you experience your daily life as an invertebrate collector? How would you describe invertebrate collection in relation to other jobs (seaweed farmers, dema fishers etc)? Are the any major problems associated with invertebrate collection?
- Let’s talk about the invertebrate collection some more. Do you sell your catch? If yes, who do you sell them to? How much money do you generally earn from invertebrates? Do you think the buyer sell the invertebrates again to a higher price? Do you think it is ok if the buyer sells them to a higher price?
- If you sell you catch, do you discuss your price with other invertebrate harvesters? Why, why not?
- Could you think of anything that would help you improve the situation?
- Is there anything stopping you from doing something else? Money, knowledge, networks?
- If you were not able to survive only on collecting invertebrates, what would you do in addition? Can you do something else? If no, why not?
- Most fishermen are involved in a fishing committee, how about you? Do you have any committee where you discuss problems and solutions?
- If not, how come you do not have a committee or belong to one? Traditional/cultural reasons? Time constraints? Support? Knowledge? Other?
- What would you need in order to organize? Money, knowledge, support etc?
- What do you think an invertebrate collection committee could do for you and other harvesters in the village? What could the benefits and disadvantages be of organizing and joining forces? How often do you think you should meet and what would you discuss?
- Would you like to have a committee? If no, why not?
- Do you think that you will continue collecting invertebrates/spear fishing in the future? Will the next generation (your children) take over after you?
- What are the most important issues to improve your life situation?
- What do you decide in your household? What does your spouse (husband/wife) decide? Who has the main responsibility for income, children, cooking etc?
- Would you like to have it any other way?
- Is there anything stopping you from making such changes?
- If you, for example would quit collecting invertebrates, could and would you be able to do that?
- Would your spouse (husband/wife) be supporting if you would like to stop and do something else, for example education/training? Would your spouse think it would be ok for you to work in some other village?
- How do you consider your role and importance in the household and society compared to your spouse (husband/wife)? How do you value your work compared to others (men and women)? Equally important? Equally valued in the society?
- Do you feel any support from the rest of the village? Why/why not?
- How would you react if your spouse were to put less time and effort into the household duties?
